# Penile Mondor Disease Following Circumcision: Diagnostic Insights from Color Doppler Ultrasound

**DOI:** 10.3390/diagnostics16030409

**Published:** 2026-01-27

**Authors:** Konstantinos Douroumis, Konstantinos Kotrotsios, Panagiotis K. Levis, Evangelos Fragkiadis, Stamatios Katsimperis, Themistoklis N. Spyridopoulos, Konstantinos Stravodimos, Napoleon Moulavasilis

**Affiliations:** 1 First Department of Urology, Medical School, National and Kapodistrian University of Athens, 11527 Athens, Greece; 2 Department of Surgery, Elpis General Hospital of Athens, 11522 Athens, Greece; 3 Second Department of Urology, Medical School, National and Kapodistrian University of Athens, 15126 Athens, Greece; 4 EchoHealth Pediatric and Adult Ultrasound Clinic, 11634 Athens, Greece

**Keywords:** penile Mondor disease, penile triplex, thrombophlebitis

## Abstract

Dorsal vein thrombophlebitis, or penile Mondor disease, is a rare benign penile condition presenting with cord-like induration at the dorsum of the penis. This induration is caused by an isolated thrombosis of the dorsal superficial vein of the penis. As symptoms are not typical and many patients are asymptomatic, it is often underdiagnosed. Causes include trauma, infection, sexual activity, genital surgery, and cancer. Differential diagnosis includes Peyronie’s disease and sclerosing lymphangitis, and diagnosis remains crucial as it facilitates the treatment plan and reassures the patient. Treatment consists of conservative measures, such as oral nonsteroidal anti-inflammatory medications (NSAIDs) and anticoagulation, and surgical management, with excision of the thrombosed vein. We present a case report of penile Mondor disease following circumcision, with the aim to provide educational ultrasound images of this rare entity. The patient, 32 years old, on the sixth postoperative day, developed a cord-like induration, along with pain, at the dorsum of the penis. Physical examination revealed a cord-like mass on the dorsal aspect of the penis. Penile triplex demonstrated a lack of endoluminal flow signals of the superficial dorsal veins, which were uncompressible. Triplex of the femoral and iliac veins showed no sign of thrombosis. Clinical presentation, along with imaging findings, facilitated the diagnosis of Mondor disease. The patient was treated conservatively with sexual abstinence and NSAIDs, and 6 weeks after the presentation, the patient was asymptomatic, without evidence of the disease in clinical examination.

**Figure 1:**
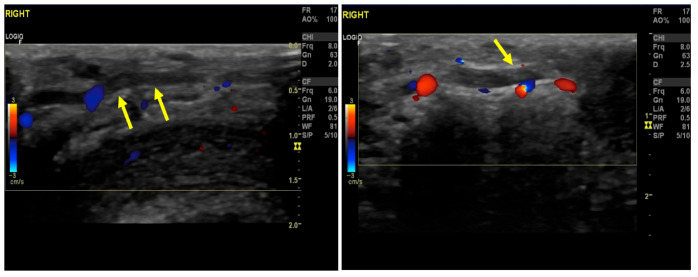
Longitudinal Color-Doppler ultrasound of the penis showing endoluminal echogenicity consisting of the thrombus (arrows), along with the absence of vascular flow signals within the dorsal vein of the penis. The examination was performed with the patient in a supine position, using a vascular linear probe (General Electric, Boston, MA, USA) (frequency range: 12–4 MHz). These images are pathognomonic and, along with the history and clinical examination, are sufficient to diagnose Mondor disease [1,2]. Despite its distinctive clinical and imaging features, Mondor disease remains under-recognized, constituting a definitive treatment algorithm impossible to be designed. The current literature supports conservative treatment, which includes NSAIDs and sexual abstinence, and reserves anticoagulation treatment for cases with proven thrombophilia or recurrent disease. Surgical excision should be considered only in refractory cases [3]. As our patient had no history of thrombophilia, sexual abstinence and pain relief management with NSAIDs were offered. After 6 weeks, the penile induration had been resolved.

**Figure 2:**
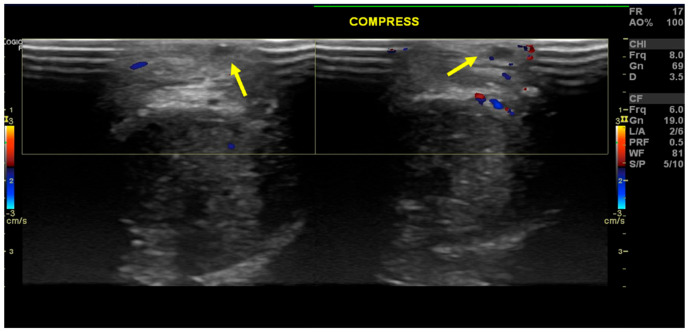
Axial Color-Doppler ultrasound of the penis. Arrows show the superficial dorsal vein. After compression of the penis, the vein remains non-compressible, another pathognomonic feature of this entity [1]. This case report is of great educational value, as many pathognomonic features of the disease are present on the ultrasound, namely the presence of endoluminal echogenicity, absent flow, and vessel incompressibility. Other characteristic findings include painful selective pressure, an increase in the caliber of the vein, and the presentation of inflammatory changes in the venous wall [1]. Differential diagnosis includes sclerosing lymphangitis and Peyronie’s disease, and is based on clinical history, physical examination, and ultrasound evaluation. Regarding ultrasonographic features, sclerosing lymphangitis presents with serpiginous (worm-like aspect) morphology, with thickened and dilated lymphatic vessels, without evidence of dorsal vein thrombosis. On the other hand, Peyronie’s disease presents as thickening of the tunica albuginea on the ultrasound, along with fibrotic plaques, which can be calcified [1].

**Figure 3:**
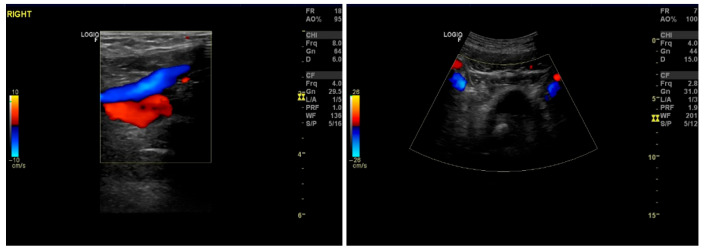
Right femoral vein (**left**) and internal iliac veins (**right**) without evidence of thrombosis. Imaging of these veins is not endorsed by the current literature, but we included it in the diagnostic work-up to ensure that the patient had solitary dorsal vein thrombophlebitis, without involvement of any major vessel, as a case has been described in the literature resulting from compression of the common iliac vein [4]. As the ultrasound of these vessels is easy to perform along with the penile ultrasound, we suggest that it should be performed in all cases of penile Mondor disease suspicion. Magnetic resonance angiography (MRA) has been reported in cases where a larger picture of the venous system is needed, such as after prostate biopsies, to exclude the possibility of hematoma, and in cases where other etiologies, such as trauma, tumors, or thrombosis of other vessels suggesting thrombophilia, have to be ruled out [1,5,6]. In our case, as the ultrasound findings confirmed the clinical suspicion of Mondor disease, an MRA was not performed.

## Data Availability

The original contributions presented in this study are included in the article. Further inquiries can be directed to the corresponding author.
